# Heat-inactivated *Streptococcus pneumoniae* augments circadian clock gene expression in zebrafish cells

**DOI:** 10.1038/s41598-024-78888-0

**Published:** 2024-11-13

**Authors:** Camila Morales Fénero, Raina E. Sacksteder, Andrew G. Diamos, Jacqueline M. Kimmey

**Affiliations:** Department of Microbiology and Environmental Toxicology, University of California Santa Cruz, Santa Cruz, USA

**Keywords:** Bacteria, Cellular microbiology, Circadian rhythms

## Abstract

The circadian clock is a cell-autonomous process that regulates daily internal rhythms by interacting with environmental signals. Reports across species show that infection can alter the expression of circadian genes; however, in teleosts, these effects are influenced by light exposure. Currently, no reports analyze the direct effects of bacterial exposure on the zebrafish clock. Using zebrafish Z3 cells, we demonstrate that exposure to heat-killed *Streptococcus pneumoniae* (HK-Spn) augments the expression of core repressive factors in a light- and time-dependent manner. In constant darkness, HK-Spn highly upregulated *cry1a*,* per3*, and *per1b* expression. In the presence of light, HK-Spn exposure rapidly and strongly upregulated *per2* and *cry1a*, and this was proportionally increased with light intensity. The combinatorial effect of light and HK-Spn on *per2* and *cry1a* was not duplicated with H_2_O_2,_ a known byproduct of light exposure. However, the ROS inhibitor N-acetyl cysteine was sufficient to block HK-Spn augmentation of *per2*, *cry1a*, and *per3*. These findings demonstrate that exposure to an inactive bacteria influences the expression of zebrafish clock genes under different light conditions.

## Introduction

Circadian rhythms are 24-hour oscillations that control different aspects of our physiology, including sleep patterns, metabolism, body temperature, hormone secretion, and immune response^1–3^. In vertebrates, these oscillations are regulated by a transcriptional/ translational feedback loop known as the molecular clock^4^. The activating arm of the molecular clock consists of the transcription factors Clock and Bmal, which dimerize and recognize promoter elements called E-boxes, leading to the circadian expression of hundreds of genes. Clock:Bmal also drives the production of the core repressive factors, Per and Cry, which inhibit Clock:Bmal activity, leading to a negative feedback loop that ultimately terminates *Per* and *Cry* expression^4^. This cell intrinsic cycle takes about 24 h to complete and is what drives daily rhythms in gene expression.

The molecular clock constantly aligns with environmental cues, such as light, to maintain synchronization with the day-night cycle^5–7^. Within vertebrates, light regulation of the clock has been extensively studied in mice, where photic input is only effective in vivo as it must be sensed through photosensitive retinal ganglion cells (pRGCs) in the eye. pRGCs signals directly activate a brain region called the suprachiasmatic nucleus (SCN), which serves as the master clock in the animal^4^. Peripheral murine cells, either in vivo or ex vivo, are unresponsive to light and instead rely on physiological cues such as body temperature, nutrients, or hormones to synchronize their timing^8^. In contrast, in zebrafish (*Danio rerio*), every cell directly detects and responds to light, whether in vivo or in vitro, making it a valuable tool for studying the vertebrate molecular clock in a whole organism or cell culture^9–11^. Zebrafish contain the same components of the molecular clock of other vertebrates (Clock, Bmal, Per, and Cry proteins); the difference between these systems stems from the fact that, in zebrafish, light drives transcriptional induction of *per2* and *cry1a* due to the presence of D-boxes in their promoters^11–18^. This light-responsive feature allows the autonomous resetting of peripheral clocks, a characteristic that is also shared by lower organisms such as *Drosophila*^19,20^. Though mammals experience a delay between the neural detection of light and the resetting of peripheral clocks^8^, light remains one of the most important environmental cues that drive circadian timing across all domains of life.

Circadian rhythms are well known to control diverse aspects of our physiology, including activity, sleep, cognition, and metabolism^3^. Circadian rhythms also drive time-of-day-dependent differences in a variety of immune pathways and have been linked to the severity of infection in humans and diverse animal models ranging from mice to *Drosophila*^2,21–28^. In humans, clock disruption due to behavioral changes such as shift work is associated with decreased health and increased risk of respiratory infection^29,30^. Moreover, vaccine efficacy can differ depending on the time of administration^31–33^. Incredibly, exposure to blue light has been reported to improve appendicitis prognosis in patients due to its ability to activate REV-ERBα, a master component of the circadian stabilization loop^34^. The first animal model demonstrating time-of-day dependent susceptibility to infection was published in 1969, finding that mice were more resistant to *Streptococcus pneumoniae* (*Spn*) during their active phase^35^. No cellular explanation for this differential bacterial control was described for over 50 years until a 2020 report demonstrated that circadian regulation of phagocytosis of *Spn* by murine macrophages is dependent on the master clock protein BMAL1^36^. Curiously, a 2012 study had already reported that in *Drosophila*, the master clock repressor *Timeless* (*tim*) differentially influences phagocytosis during day and night, regulating resistance against *Spn*^28^. These findings suggest a strategy that may be conserved across species despite the partial divergence of the core clock proteins, whereby the circadian phase dictates how cells respond to pathogens.

Dissecting these cellular mechanisms is complicated because substantial evidence demonstrates that active infection can directly alter the expression of the circadian clock. Infection with *Helicobacter pylori* has been shown to upregulate the expression of *Bmal1* in both murine and human cells^37^. In contrast, there are many more examples of infection downregulating clock genes. Human cells infected with the hepatitis C virus have decreased PER2 and CRY2, and mice infected with the eukaryotic parasites *Trypanosoma brucei* and *Plasmodium chabaudi* have decreased *Bmal1*, *Per1*, and *Dbp*^38,39^. In zebrafish, RNA-seq analysis revealed that infection with the microsporidium *Pseudoloma neurophilia* downregulated the expression of multiple genes related to circadian rhythms in the fish brain, including *per1b* and *nr1d1*^40^. Not surprisingly, several reports in teleosts demonstrate that light exposure is a critical variable that impacts how clock genes respond during infection, innate immune activity, and ultimately, the survival of zebrafish during bacterial infection^41–44^. Finally, another limitation is that these studies do not address whether changes in circadian gene expression result from the pathogen directly (e.g. detection of microbial ligands, secretion of virulence factors), inflammation, or host tissue damage. Several reports have shown that, at least in murine models, exposure to pathogen-associated molecular patterns (PAMPs) alone can alter circadian gene expression, most commonly resulting in decreased amplitude of expression^45–51^. However, no studies have investigated whether microbial ligands affect circadian gene expression in zebrafish.

Considering this, this study aimed to characterize the impact of exposure to an inactivated Gram-positive bacterium, *Streptococcus pneumoniae*, on the expression of two light-responsive clock genes from the repressive arm: *per2* and *cry1a*, and two non-light-responsive genes, *per1b* and *per3*, in zebrafish cells.

## Results

### Basal and light-induced expression of circadian genes on Z3 cells

**Fig. 1:**
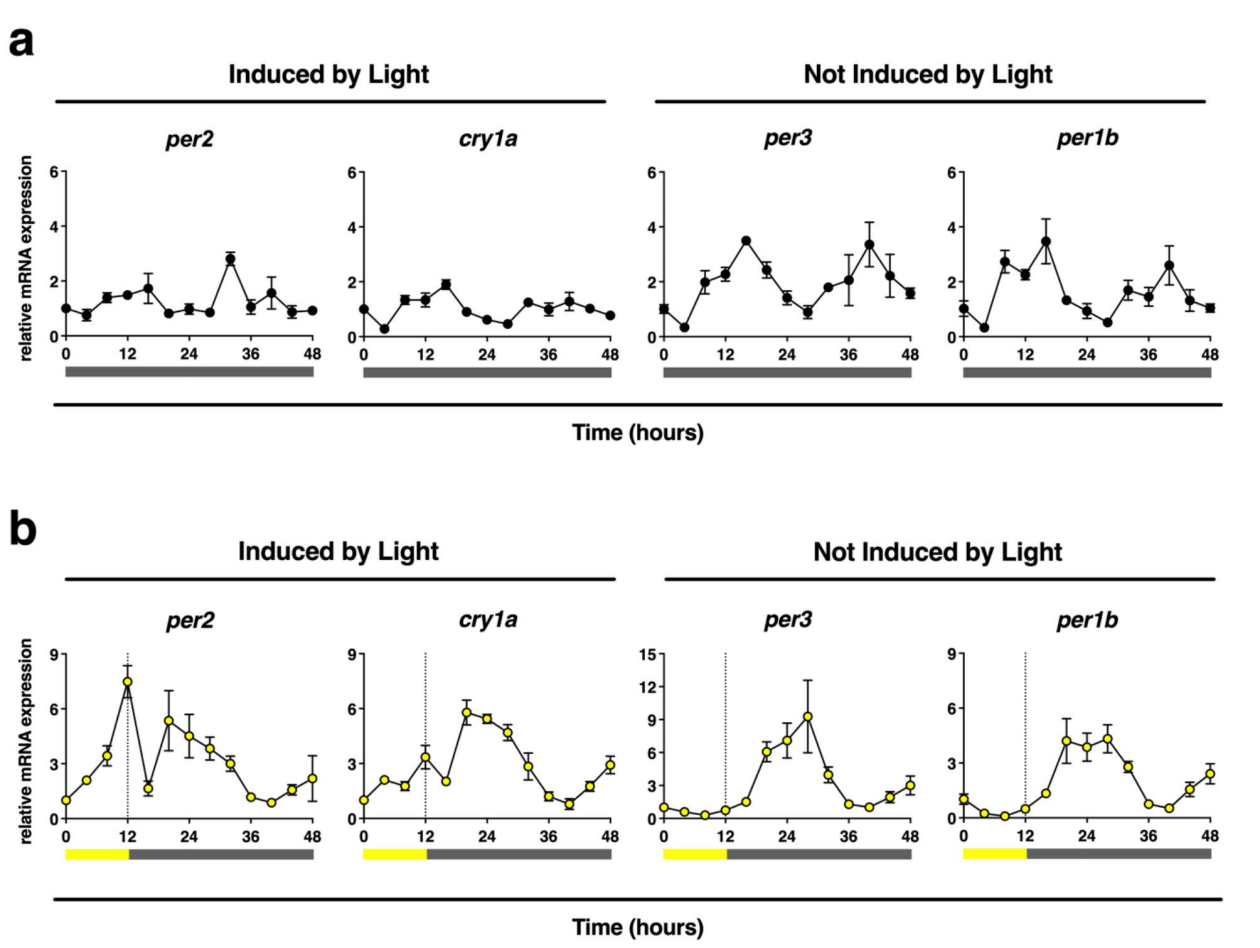
Effect of light exposure in the expression of *per2*, *cry1a*, *per3*, and *per1b* on Z3 cells. (**a**) Z3 cells were entrained for three LD cycles of 12:12 h. After the third day of entrainment, cells were exposed to constant darkness (DDDD) and mRNA expression of the genes *per2*, *cry1a*, *per3*, and *per1b* were analyzed every 4 h for 48 h. The eJTK test detected circadian rhythmicity on *per3* and *per1b*, with a *p*-value of 0.01. No rhythmicity was detected in *per2* and *cry1a* in DDDD. (**b**) mRNA expression of the genes *per2*, *cry1a*, *per3* and *per1b* after a 12-hour exposure of low-intensity light (~ 4.12 × 10^18^ photons/s/m^2^) with subsequent darkness (LDDD). Samples were taken every 4 h for 48 h. Transcript levels were measured against time zero. Yellow bar: light exposure. Grey bar: constant darkness. Means ± SD are shown. Three replicates per group, one experiment.

In the last two decades, numerous studies have demonstrated the importance of light regulating the zebrafish clock^10–18,52–60^. Light activates the transcription of the clock repressors *per2* and *cry1a*, directly resetting the phase of the clock^11,14–18^. Because different light conditions and circadian phases of exposure have been shown to have varying induction levels on *per2* and *cry1a* expression^15,58,59^, we first sought to characterize these genes by a qRT-PCR time course in our cells. We choose *per3* and *per1b* as control genes not induced by light^11,60^. We first entrained Z3 cells, a zebrafish embryonic fibroblast-like cell line^11^, for at least three days in 12 h light and 12 h dark (12:12 LD, low-intensity white light ~ 4.12 × 10^18^ photons/s/m^2^). To assess the self-sustained circadian rhythm, cells were then maintained in continual darkness (DDDD) for 48 h, during which time we analyzed the transcripts expression of *per2*,* cry1a*,* per3*, and *per1b* every 4 h (Fig. 1a). Consistent with previous literature^11,53^, *per2* and *cry1a* show minimal oscillations in constant darkness. To determine circadian rhythmicity, we analyzed the expression of these genes in DDDD, using the JTK algorithm on BioDare2 software. This is a non-parametric test that is widely used in circadian rhythm studies to detect oscillating molecules. While *per2* and *cry1a* appear to oscillate in the dark, neither of these genes passed the JTK rhythmicity test. In contrast, *per1b* and *per3* showed robust and significant oscillations in the dark, confirmed by a positive JTK rhythmicity test.

To validate the effect of light on the induction of *per2* and *cry1a*, we repeated these studies with an additional 12-hour light exposure during the first 12 h of analysis (Fig. 1b). As predicted, light drove immediate increases in *per2* and *cry1a* (0–12 h), but not to *per1b* and *per3*, which do not respond to light. After light exposure, all four genes maintained oscillations in the following 36 h of darkness. These data demonstrate that the light-regulated expression patterns of these genes, previously shown in other studies^11^, are maintained in our experimental conditions.

### Heat-killed *Streptococcus pneumoniae *augment gene expression of the zebrafish clock’s repressive arm

**Fig. 2:**
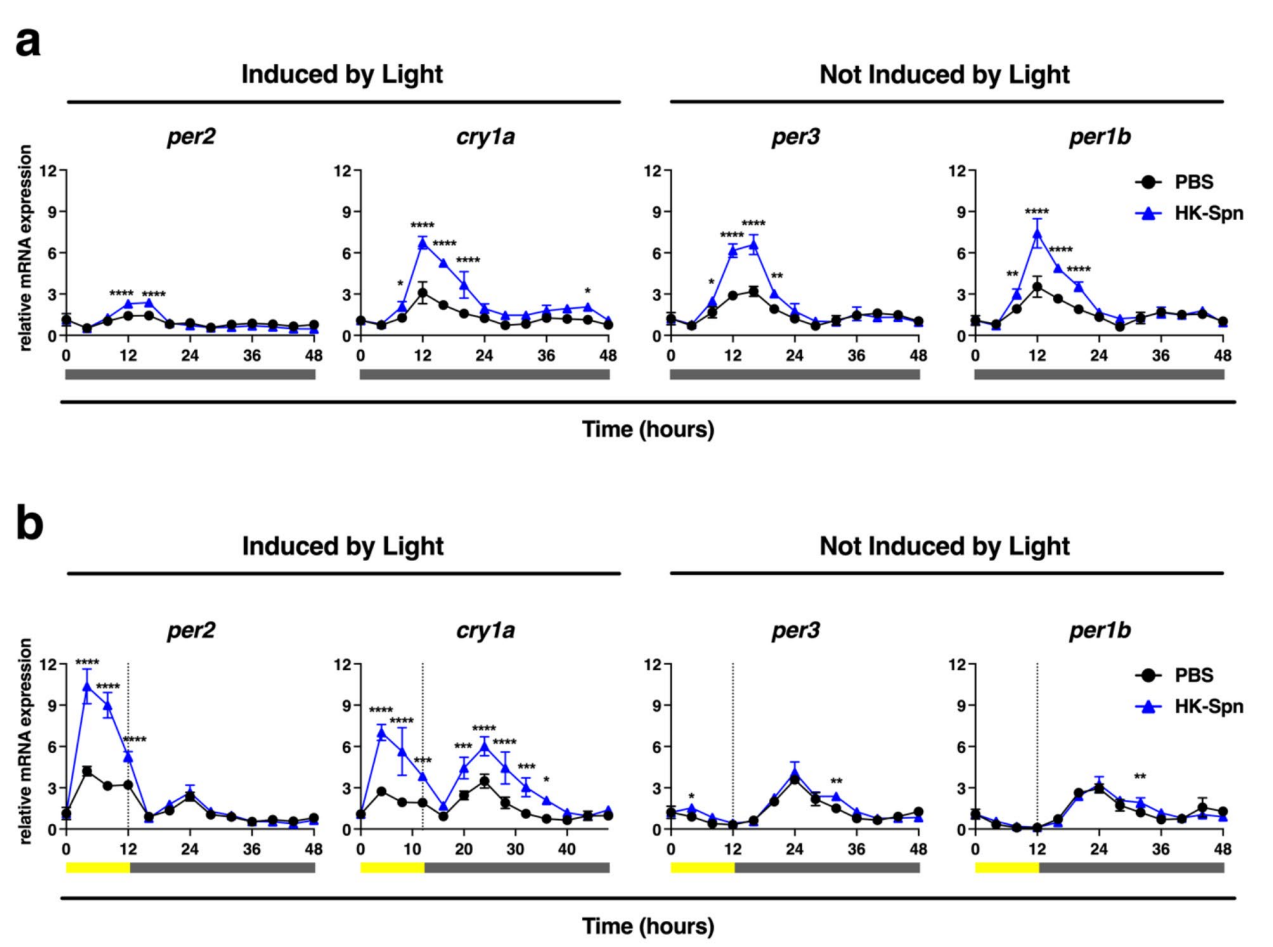
HK-Spn exposure augments the expression of *per2*, *cry1a*, *per3*, and *per1b *on zebrafish cells. (**a**) After entrainment, Z3 cells were exposed to PBS or heat-killed *Streptococcus pneumoniae* (HK-Spn) and maintained in constant darkness (DDDD). RNA samples were collected every 4 h throughout 48 h, and the relative transcript expression of *per2*, *cry1a*, *per3*, and *per1b* was measured. (**b**) Z3 cells were exposed to PBS or heat-killed *Streptococcus pneumoniae* (HK-Spn) under 12-hour low-intensity light (~ 4.12 × 10^18^ photons/s/m^2^) followed by constant darkness (LDDD). RNA samples were collected every 4 h throughout 48 h, and the relative transcript expression of *per2*, *cry1a*, *per3*, and *per1b* was measured. All time points were compared against time zero samples. Yellow bar: light period. Grey bar: dark period. Means ± SD are shown. Asterisks (*) indicate statistically significant differences *p < 0.05,** *p* < 0.01,***p < 0.001, ****p < 0.0001, Two-way ANOVA with Šídák’s multiple comparisons test. Three replicates per group, one experiment.

*Streptococcus pneumoniae* (*Spn*) is a Gram-positive pathogen that has been shown to elicit a circadian-dependent host response when infecting different organisms, including mice and *Drosophila*^28,35,36^. However, no studies have shown whether the sole presence of this bacteria directly affects the clock in zebrafish. Therefore, we wondered if exposure to an inactive form of *Spn* affects the expression of zebrafish clock genes. To test this, we incubated Z3 cells with heat-killed *Streptococcus pneumoniae* (HK-Spn) and exposed them to the same light conditions that had been tested previously. In constant darkness, HK-Spn substantially augmented the amplitude of *cry1a*,* per3*, and *per1b* oscillations within the first 24 h of exposure (Fig. 2a). In contrast, a small but statistically significant augmentation of *per2* did occur in response to HK-Spn at 12 and 16 h. Importantly, the gene expression pattern of these genes in the presence of HK-Spn does not show any changes in phase but rather augments the natural expression of these genes.

Because several reports in teleost have shown that light plays a differential role in how the clock interacts with microbes, we decided to analyze the exposure to HK-Spn in the context of light. As in Fig. 1, immediate increases in *per2* and *cry1a* occurred in response to light, but surprisingly, exposure to HK-Spn further amplified the light-induced expression of *per2* and *cry1a* (Fig. 2b). Moreover, the amplitude of *cry1a* expression was also augmented during the subsequent dark period (20–36 h), while *per2* was not affected. In contrast, there was no substantial increase in amplitude in *per3* and *per1b* expression in this condition, but small significant differences were seen at 4 (*per3*) and 32 h (*per3* and *per1b*). Together, these results demonstrate that the sole presence of bacteria, in the absence of an infection, is sufficient to affect the expression of light-inducible (*per2*, *cry1a)* and non-inducible (*per3*, *per1b)* clock genes. Moreover, the expression patterns of HK-Spn augmentation follow the natural expression of these genes, although the effect seems to depend on light conditions present during bacteria exposure.

### HK-Spn acutely augments *per2*, *cry1a*, and *per3* in different light conditions

**Fig. 3:**
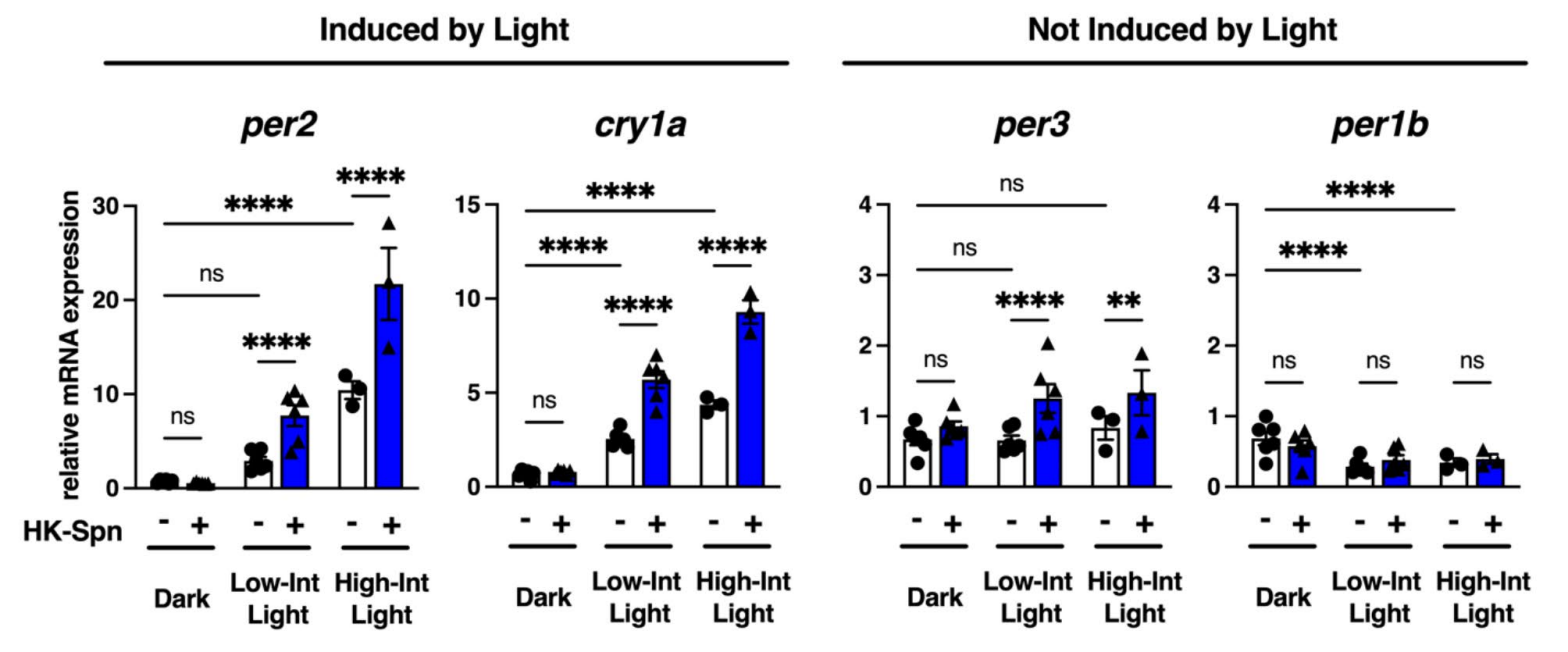
Light intensity proportionally augments HK-Spn effect on *per2 *and *cry1a *but not in *per3 *and *per1b*. Z3 cells were entrained and exposed to HK-Spn under two light conditions, low-intensity light (Low-Int light,~4.12 × 10^18^ photons/s/m^2^) and high-intensity light (High-Int light, ~ 3.96 × 10^19^ photons/s/m^2^) for 4 h. Transcript expression of *per2*, *cry1a*, *per3*, and *per1b* was compared against time zero samples. Means ± SEM are shown. Asterisks (*) indicate statistically significant differences ** *p* < 0.01,****p < 0.0001, Two-way ANOVA with Tukey’s multiple comparisons test. Six experiments were conducted for dark and low-intensity light conditions, and three were conducted for high-intensity light conditions. Three replicates per group.

Given that *per1b*, *per2*, *per3*, and *cry1a* are part of a negative feedback loop that can affect their own expression, we decided to focus on the acute induction of genes within the first 4 h of exposure. At this time point, the changes observed are most likely to stem from the direct effects of HK-Spn exposure. Because the intensity of light affects the expression of *per2* and *cry1a*^14,18^, and since the most prominent impact of HK-Spn exposure was seen in *per2* light-driven expression (Fig. 2b), we next sought to determine whether HK-Spn would have the same effect at different light intensities. At 4 h, HK-Spn is insufficient to induce *per2* or *cry1a* expression in the dark (Fig. 3). As in Fig. 2b, low-intensity light (~ 4.12 × 10^18^ photons/s/m^2^) induces *per2* and *cry1a* expression (~ 3-fold and ~ 2.5-fold on average, respectively), which is further augmented in the presence of HK-Spn (~ 7.7-fold and ~ 4.7-fold on average, respectively). Notably, *per2* and *cry1a* induction is strongly increased with high-intensity light (~ 3.96 × 10^19^ photons/s/m^2^; ~10.4-fold and ~ 4.4-fold, respectively), which is further magnified in the presence of HK-Spn (*per2* ~ 21.7-fold and *cry1a* ~ 9.3-fold). These results show that immediately following HK-Spn exposure, *per2* and *cry1a* are proportionally augmented by increasing light levels, but darkness impairs this acute activation.

In the case of *per3*, neither low-intensity light, high-intensity light, nor HK-Spn alone was sufficient to induce expression (Fig. 3). However, when HK-Spn was added in the presence of light at either intensity, there was a significant effect (~ 1.3-fold increase). In contrast, *per1b* shows diminished expression during light exposure, consistent with reports that show inhibition by light^60^. Moreover, HK-Spn did not augment *per1b* expression at this time point, as seen in Fig. 2. These results suggest that light is needed for HK-Spn acute effects on *per2*,* cry1a* and *per3.* Hence, we propose that a common transcriptional activating factor generated by light allows HK-Spn-induced acute augmentation.

### Exogenous H_2_O_2 _does not replicate light and HK-Spn combinatorial effects

Previous reports have described that light drives the expression of *per2* and *cry1a* through the activation of D-box regions in their promoters^16–18^. Light induction of *per2* and *cry1a* has also been related to the production of reactive oxygen species (ROS) by different light wavelengths, an effect mediated by activation of MAP kinases and D-boxes^52,53^. Moreover, H_2_O_2_ is sufficient to induce the expression of *per2* and *cry1a*, which has also been suggested to occur through the activation of D-boxes^52,53^. In line with this, we wondered whether H_2_O_2_ is sufficient to mimic the effect of light and HK-Spn augmentation. To test this, we exposed Z3 cells to 300 µM H_2_O_2_ alone or in combination with HK-Spn in dark conditions and monitored gene expression after 4 h.

**Fig. 4:**
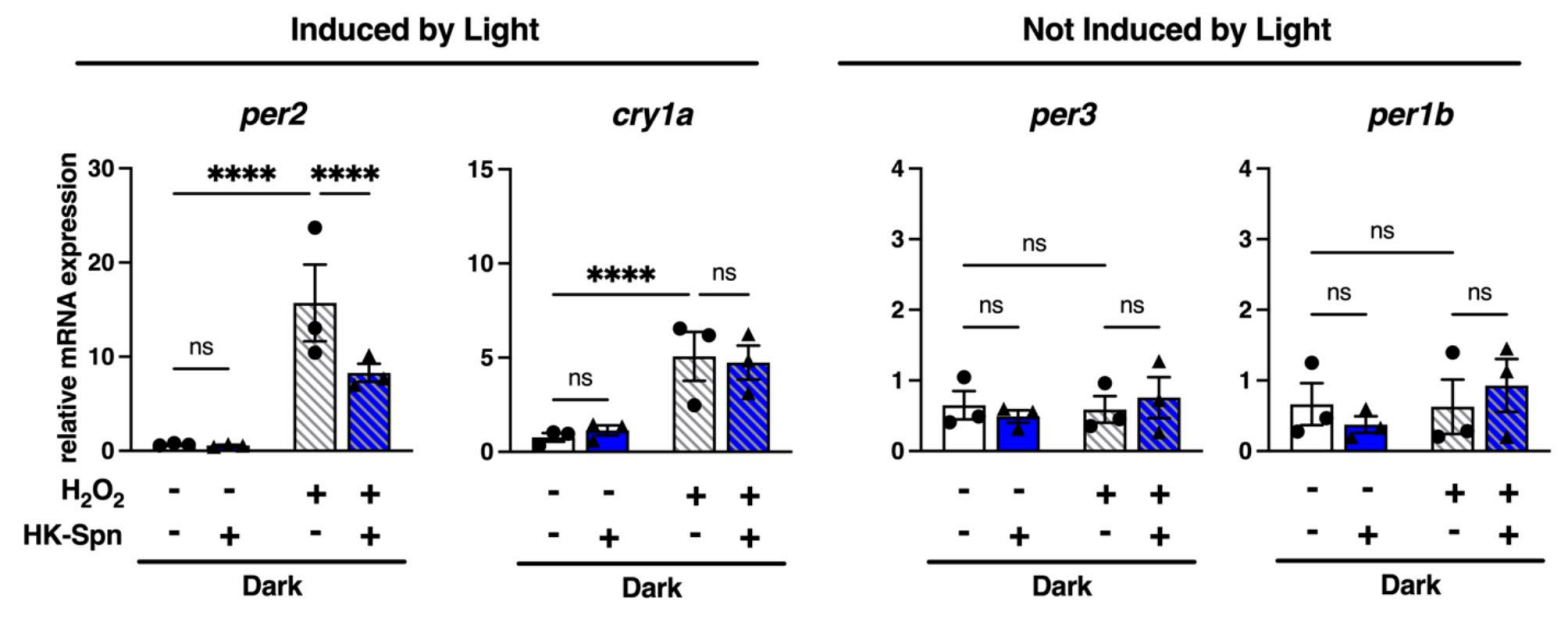
Hydrogen peroxide does not replicate light and HK-Spn effects. Relative expression of *per2*, *cry1a*, *per3*, and *per1b* in Z3 cells incubated with 300 µM hydrogen peroxide (H_2_O_2_) and HK-Spn for 4 h in the dark. Means ± SEM are shown. Asterisks (*) indicate statistically significant differences ****p < 0.0001, Two-way ANOVA with Tukey’s multiple comparisons test. Three experiments with three replicates per group.

As expected, H_2_O_2_ induced robust expression of *per2* and *cry1a* (Fig. 4). However, contrary to light, there was no combinatorial effect with HK-Spn. Moreover, H_2_O_2_ was not sufficient to induce *per3* or *per1b*, alone or in combination with HK-Spn. This is consistent with previous reports indicating H_2_O_2_ activates D-boxes, regions absent in *per1b* and *per3*^52^. These results indicate that H_2_O_2_ does not recapitulate the combinatorial effect of light and HK-Spn on *per2*,* cry1a*, and *per3*.

### Role of ROS in HK-Spn mediated effect on *per2*, *cry1a*, and *per3 *expression

**Fig. 5:**
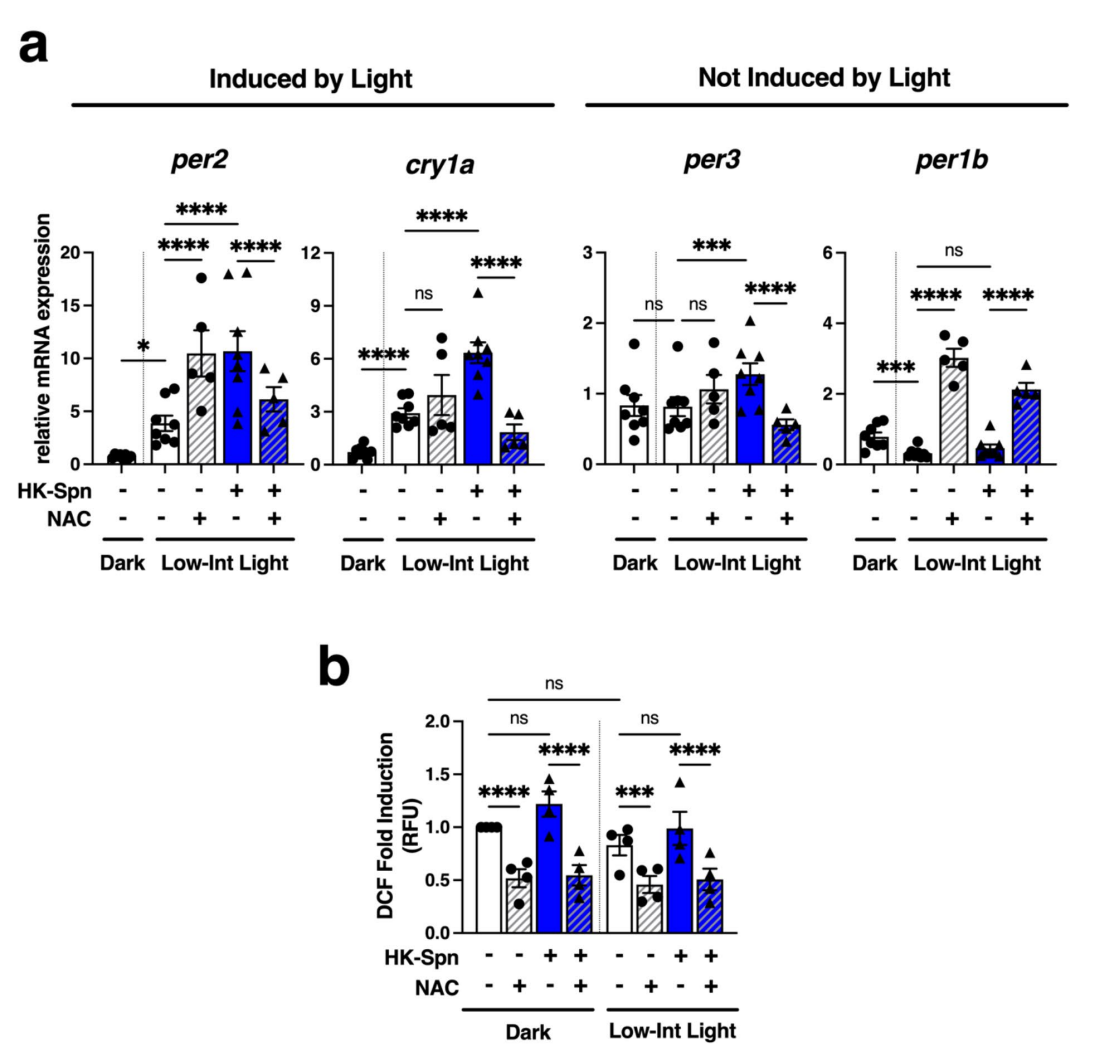
Role of ROS in HK-Spn and light induction of clock genes. (**a**) Relative expression of *per2*, *cry1a*, per3, and *per1b* of Z3 cells exposed to HK-Spn and incubated in dark and low-intensity light conditions for 4 h. A 2-hour pre-treatment of 6 mM of N-acetyl cysteine (NAC) was used to suppress ROS production. Means ± SEM are shown. Asterisks (*) indicate statistically significant differences *p < 0.05, ***p < 0.001, ****p < 0.0001, Two-way ANOVA with Tukey’s multiple comparisons. Five to eight experiments, three replicates per group. (**b**) Dichlorodihydrofluorescein (DCF) fluorescence was measured after 2 h of PBS or HK-Spn exposure in dark and low-intensity light conditions. A 2-hour pre-treatment of 6 mM of N-acetyl cysteine (NAC) was used to suppress ROS production. Fold induction of relative fluorescent units was calculated against the dark PBS-treated cells. Means ± SEM are shown. Asterisks (*) indicate statistically significant differences ***p < 0.001,****p < 0.0001, Two-way ANOVA with Tukey’s multiple comparisons test. Four experiments, three replicates per group.

Even though H_2_O_2_ was not sufficient to induce a combinatorial effect with HK-Spn, we wondered if the light-generated ROS may activate alternative pathways from exogenous H_2_O_2_ and thus contribute to the augmentation effect of HK-Spn. To test this, we treated Z3 cells with the antioxidant N-acetylcysteine (NAC) and evaluated gene expression at 4 h in light and dark conditions. NAC was not sufficient to inhibit light-mediated induction of *per2* and *cry1a* (Fig. 5a), but instead, it significantly decreased the effect of HK-Spn augmentation of *per2*, *cry1a*, and *per3*. These results lead us to hypothesize that ROS generated in response to HK-Spn may be responsible for the augmentation of *per2*,* cry1a*, and *per3*. Unexpectedly, NAC induced *per1b* expression even in the absence of HK-Spn, suggesting that *per1b* expression may be sensitive to redox balance in a distinct manner.

Because the innate immune detection of bacterial components often results in ROS production, we hypothesized that Z3 cells might generate ROS in response to HK-Spn exposure, contributing to the augmentation of *per* and *cry* genes. To test this, we analyzed the generation of ROS by the DCFH-DA (DCF) assay. DCFH-DA is a cell-permeable reagent that gets trapped in cells upon cleavage by intracellular esterases and fluoresces upon oxidation by intracellular ROS^61,62^. A pilot study to identify the best timepoint for analysis was conducted to analyze ROS production at 0.5, 1, 2, and 4 h post stimulation with HK-Spn. All timepoints showed similar patterns (i.e., fold differences between groups were similar at all timepoints) though, as reported previously in the literature, we also observed general increases in DCF baseline signal that correlates with incubation time. As we reasoned that changes in ROS would have to precede the activation of gene expression, we selected the 2-hour timepoint to analyze with sufficient replicates. Thus, after 2 h of stimulation with HK-Spn and LD exposure, cells were incubated with DCF, washed, and the fluorescent signal was measured at 490 nm excitation and 530 nm emission wavelengths. Fold induction was calculated against the basal levels of ROS detected in the dark (Fig. 5b). NAC, which inhibits ROS production, was used as a negative control. Exposure to light for 2 h did not induce a significantly higher DCF signal when compared to dark. Similarly, HK-Spn did not show detectable changes in the DCF-ROS signal in either dark or light conditions, but NAC treatment decreased ROS levels in all conditions. These results suggest that light and HK-Spn do not generate higher ROS production detectable by DCF in our conditions, suggesting NAC’s effects may be independent of ROS.

## Discussion

Several studies have demonstrated changes in circadian gene expression during infection with bacteria, viruses, and parasites^37–40,42–44^. However, one question that has remained unanswered is whether this is due to direct effects of bacteria (or lack of bacteria) or indirect effects such as inflammation and damage from an infection. In this study, we demonstrate for the first time that the presence of inactive bacteria, specifically the Gram-positive *Streptococcus pneumoniae*, can induce changes in the expression of zebrafish genes from the repressor arm of the circadian clock suggesting that vertebrate hosts perceive and react to the presence of microbes. This finding is intriguing as our current models of circadian clocks do not provide any mechanistic reasons as to how or why the detection of bacteria would affect the core timekeeping machinery. Interestingly, these gene-specific effects are affected by the light conditions and the temporal expression of these genes. Here we show that heat-killed *Streptococcus pneumoniae* potently augmented the expression of *per2* and *cry1a* in a manner synergistic with light and independently augments *cry1a*,* per3*, and *per1b* in constant darkness. This effect is surprising as several studies have demonstrated that exposure to bacterial PAMPs decreases the expression of clock genes^38–40,45–51,63,64^. In line with this, inflammatory compounds also decrease the amplitude of *per3* oscillations in larval zebrafish^65^. It is clear that inflammatory and microbe recognition pathways can affect the circadian clock but these effects may vary depending on the time of exposure, the type of microorganism, and environmental conditions^39,50,51,65,66^.

Our results demonstrate different effects on clock gene expression depending on light exposure, an effect that is consistent with previous studies. In rainbow trout (*Oncorhynchus mykiss*), a skin infection with the ectoparasite *Argulus foliaceus* altered the expression and rhythmicity of clock genes in a manner dependent on light conditions^42^. Moreover, fish infected and maintained in constant light conditions had more parasite load after seven days of infection than those exposed to regular LD cycles. In a zebrafish model infected with tilapia lake virus, light availability significantly enhanced the antiviral immune response and changed the gene expression of clock genes (e.g., upregulation of *arntl2* in the kidney in LD conditions)^43^. In a recent study, infection of zebrafish larvae with the gram-negative *Klebsiella pneumoniae* showed a general trending of upregulated clock gene expression in the light but downregulation in the dark^44^. These studies demonstrate that changes in clock expression are specific to both the pathogen and the light context during exposure, having consequences that can influence the disease outcome.

To investigate the combinatorial effects seen between light and HK-Spn exposure, we also examined the contribution of ROS in this pathway. Several reports have demonstrated a bidirectional interaction between ROS and clock genes in *Drosophila*, zebrafish, and mammalian systems^48,52,53,67–73^. Moreover, light-generated ROS have been related to the light-driven expression of *per2* and *cry1a* in zebrafish^52,53^. However, our results showed that in contrast to light, there is no synergistic effect between H_2_O_2_ and HK-Spn. This, together with the fact that low-intensity white light did not produce detectably higher amounts of ROS and light induction of *per2* and *cry1a* was not reduced by a ROS inhibitor, indicates that in our conditions, light-generated ROS do not play a role in *per2* and *cry1a* induction.

An intriguing fact is that the addition of the antioxidant NAC prevented HK-Spn from inducing *per2*, *cry1a*, and *per3*, suggesting a ROS-activated pathway in HK-Spn augmented response. Nonetheless, we were unable to detect a higher ROS signal in the presence of HK-Spn. A possible explanation could be that HK-Spn exposure generates a type of ROS not detectable by DCF as this assay presents a limitation in detecting different ROS species. DCF is oxidized by various radicalized ROS, including peroxyl, alkoxyl, hydroxyl, carbonate, nitrogen dioxide, and peroxynitrite, but can only detect cellular peroxides, such as H_2_O_2_, if they are decomposed to radicals^61^. Alternatively, the effects of NAC may be a consequence of other characteristics of this compound, such as a reducing agent, inhibitor of proteasome inhibitors, and suppressor of the transcription factor NF-κB, any of which may affect gene expression^74–76^. In PAC-2 cells, NAC was shown to block *per2* and *cry1a* expression induced by blue light^52^; however, here we observed that in Z3 cells, NAC has no effect on light-induced *cry1a* and can even augment expression of *per2*. These differences could possibly be attributed to cell-specific effects and/or the use of different light wavelengths. Interestingly, the addition of NAC alone was sufficient to drive *per1b* expression in the light, an effect that has not been previously reported. It is uncertain if this effect results from NAC alone or a combinatorial effect of NAC and light, but it supports the idea that complex interactions exist between redox regulation and the circadian clock that remains unexplored.

The current understanding of the transcriptional regulation of *per* and *cry* genes in zebrafish is through promoter elements called E-boxes and D-boxes, driven by clock-related transcription factors such as Clock:Bmal and Tef, respectively^12,13^. Our initial thought about the regulation of HK-Spn effects was the activation of these regions by modulation of core components of the clock; however, we cannot discount the possibility that non-circadian transcription factors contribute to these effects. In mammals, transcription factors activated by microbial exposure, such as HIF-1⍺ and NF-κB, have been shown to directly interact with E-boxes or modify CLOCK:BMAL interaction with these regions, respectively^77–82^. Future studies analyzing the contribution of circadian and non-circadian transcription factors activated by HK-Spn may answer these questions. Additionally, other elements such as promoter architecture, such as the number of spacing of binding sites, or interactions between factors, as well as post-transcriptional modifications, could be at play in this regulation^83–85^. Other questions to be addressed in future studies will investigate the nature of the heat-resistant ligand from Spn, the effect of the exposure to other types of bacteria, and the identification of the receptors that contribute to this outcome. Regardless, the findings presented in this work provide new insights into the regulatory pathways of zebrafish circadian genes and present, for the first time, the impacts of an inactive bacteria on the molecular clock of the zebrafish. Understanding the interactions between different types of bacteria and the circadian clock can provide valuable insights into the intricate relationship between these systems.

## Methods

### Z3 cell culture

Z3 cells, a fibroblast-like embryonic cell line from zebrafish (*Danio rerio*)^11^, were maintained at room temperature in Z3 Media: Leibovitz L-15 medium (Gibco, 11415064) supplemented with 15% HyClone Fetal Bovine Serum (Cyvita, 16777-238), 2µM L-Glutamine (Sigma, G3126) and 1% Penicillin/Streptomycin (Sigma, P4333). Cells were seeded at 1 × 10^6^, 5 × 10^5^, or 5 × 10^4^ cells/well into 6-, 12- or 96-well plates, respectively, grown until confluent and entrained in 12:12 LD cycle for three days using low-intensity white light (LED, 400–700 nm, ~ 4.12 × 10^18^ photons/s/m^2^). After entrainment, cells remained in the dark unless light was noted in the figure legend.

### Light sources

Cells in light conditions were exposed to low-intensity (~ 500 lux, ~ 4.12 × 10^18^ photons/s/m^2^) or high-intensity (5000 lux, ~ 3.96 × 10^19^ photons/s/m^2^) white light (LED, 400–700 nm) for 4–12 h, as noted in the figure legend. Cells in dark conditions were kept at room temperature in constant darkness and covered in aluminum foil. Treatments of all groups and harvest of the dark condition groups were performed under indirect dim red light (LED, 600–750 nm, ~ 0–2 lux, ~ 1.67 × 10^16^ photons/s/m^2^), which does not induce *per2*.

### Heat-killed bacteria

Heat-killed *Streptococcus pneumoniae* (HK-Spn) was prepared by growing wild-type D39 in Todd-Hewitt Broth (Thermo Scientific, CM0189B) with 2% Yeast Extract (Sigma, Y1625) at 37 °C with 5% CO_2_ until they reached an OD_600_ of 0.4. Colony-forming units were enumerated by serial dilution and plating on Columbia Blood Agar (Thermo Scientific, OXCM331B) with 5% Sheep’s Blood (Fisher Scientific, 50863755), and the remaining bacteria were then pelleted, resuspended in PBS, and heated to 100 °C for 1 h. HK-Spn was stored in aliquots at -20 °C until use. The effective dose of HK-Spn was determined empirically based on augmentation of *per2* and *cry1a* after 4 h in light conditions. For all experiments shown here, the actual dose used was between 3.7 × 10^8^ - 2.25 × 10^9^ CFU/mL.

### Bacterial exposure and pharmacological treatments

PBS, HK-Spn, and 300 µM H_2_O_2_ (Sigma, H1009) were applied after 12 h of the last entrainment cycle (time zero). N-Acetyl-L-cysteine (NAC) (Sigma, A9165) was added 2 h before time zero to a final concentration of 6 mM. All treatments were added directly to the cell media under dim red light. No media changes were made after entrainment.

### RNA extraction and qRT-PCR

Cells were harvested in DNA/RNA Shield (Zymo, R110050) by scraping, flash-frozen in liquid nitrogen, and stored at -80 °C until extraction. The following products were used for qRT-PCR analysis: Quick-RNA Miniprep Kit (Zymo, R1055), iScript cDNA Synthesis (Bio-Rad, 1708891), PowerUp SYBR Green (Applied Biosystems, A25777), CFX Connect Real-Time thermocycler (Bio-Rad). Primers: *actb1 (NM_131031)* Fwd: ATCTTCACTCCCCTTGTTCAC; Rev: TCATCTCCAGCAAAACCGG, *per1b (NM_212439)* Fwd: TGCGCGTAATGGAGAGTATATG; Rev: CTTCGTTCAGTGGAGAGGTTC, *per2 (NM_182857)* Fwd: ACGAGGACAAGCCAGAGGAACG; Rev: GCACTGGCTGGTGATGGAGA, *per3 (NM_131584)* Fwd: CAAGTACAAGCAAACAGCGAG; Rev: ACTACCACAAAAGAGTCCGTG, *cry1a (NM_001077297)* Fwd: GGAGTGTGAACGCAGGAAG; Rev: AAACCCCTTAAGACTGGCAG. ΔΔCT was used to calculate relative mRNA expression^86^. Values were normalized to *actb1* and compared to time zero samples.

### Rhythmicity test

An eJTK rhythmicity test was performed on 48 h gene expression of *per1b*,* per2*,* per3*, and *cry1a* in constant darkness, using the online software BioDare2 (https://biodare2.ed.ac.uk/)^87^. Data were not detrended and were analyzed with the following settings: BD2 eJTK test method, and eJTK Classic analysis presets. *p*-value of < 0.01 was considered statistically significant.

### Measurement of intracellular ROS

Cells were stimulated at time zero under dim red light, then maintained at room temperature in the dark or exposed to low-intensity light for 2 h. The following steps were all performed under dim red light: After treatments, cells were washed once with Hanks Balanced Salt Solution (HBSS) (Gibco, 14025092) and incubated for 45 min in 10 µM 2′,7′-Dichlorodihydrofluorescein diacetate (DCFH-DA) (Sigma, D6883). Cells were washed once with HBSS to remove extracellular DCFH-DA and placed in plain HBSS to read fluorescence at 490 nm excitation and 530 nm emission wavelengths using a Spectramax iD3 Multi-Mode Microplate Reader (Molecular Devices). Fold change was calculated by dividing the value of each sample by the average of the PBS dark control.

### Statistical analysis

All experiments were performed thrice with three replicates unless denoted in the figure legend. Two-way ANOVA analyzed differences between two groups at different time points with Šídák’s multiple comparisons test. Two-way ANOVA with Tukey’s multiple comparisons was used to compare more than two groups and conditions.

## Data Availability

Data is available from the corresponding author upon reasonable request.
